# Heavy metal contamination in freshwater habitats impairs the growth and reproductive health of wild spotted snakehead *Channa punctata* (Channidae) in Bangladesh

**DOI:** 10.1016/j.heliyon.2025.e42543

**Published:** 2025-02-07

**Authors:** Imran Parvez, Sharmin Ahmed, Nazifa Tasnim, Rubaiya Pervin, Md Ashraful Alam, Md Nasir Khan, Yeasmin Ara, Harunur Rashid, Siriporn Pradit

**Affiliations:** a Faculty of Environmental Management, Prince of Songkla University, Hat Yai, 90110, Songkhla, Thailand; b Department of Fisheries Biology and Genetics, Hajee Mohammad Danesh Science and Technology University, Dinajpur, 5200, Bangladesh; c Department of Fisheries Management, Hajee Mohammad Danesh Science and Technology University, Dinajpur, 5200, Bangladesh; d Department of Fisheries Management, Bangladesh Agricultural University (BAU), Mymensingh, 2202, Bangladesh; e Marine and Coastal Resources Institute, Faculty of Environmental Management, Prince of Songkla University, Hat Yai, 90110, Songkhla, Thailand; f Coastal Oceanography and Climate Change Research Center, Faculty of Environmental Management, Prince of Songkla University, Hat Yai, 90110, Songkhla, Thailand

**Keywords:** Freshwater habitat, Pollution, *Channa punctata*, Heavy metals, Reproduction, Growth

## Abstract

Heavy metal bioaccumulation in aquatic organisms of open water aquatic ecosystems was detected globally, including Bangladesh. This study evaluated the hypothesis of whether heavy metal contamination in aquatic habitats impacts fish growth and reproduction using wild *Channa punctata* as an experimental animal. The growth and reproductive health of a wild freshwater fish, *C. punctata*, collected from five freshwater habitats, were assayed with heavy metal bioaccumulation. Atomic absorption spectrometry detected the bioaccumulation of cadmium (Cd), chromium (Cr), mercury (Hg), and lead (Pb) in the muscle of *C. punctata*. Cd, Cr, and Pb concentrations were the highest in the specimen collected from the Turag River and the lowest in the Dharla River. The highest concentration of Hg was found in *C. punctata* specimens collected from the Karatoya River (0.093 ± 0.004 mg/kg). The length-weight relationship and condition factor of *C. punctata* indicated a negative allometric growth pattern (*b* < 3.0) and poor wellness (F < 1.0) in all the stocks except Dharla River. We estimated the size at first sexual maturity (L50), ova diameter, fecundity, and gonadosomatic index (GSI) to assess reproductive health and determined the correlation with heavy metal bioaccumulation. We found that higher bioaccumulation of heavy metal impairs the reproductive health of *C. punctata* by lowering spawning performance. This study showed that heavy metal bioaccumulation impaired fish's growth and reproductive health, potentially affecting future recruitment and fishery sustainability.

## Highlights

• Freshwater fish habitats of Bangladesh are being polluted with heavy metals.
• Heavy metal bioaccumulation in *C. punctata* of fresh water habitats were detected.
• Growth pattern and reproductive performances of *C. punctata* varied among the habitats.
• Heavy metal pollution impair growth and reproductive health of *C. punctatus*.

## 1 Introduction

Industries in developing nations like Bangladesh typically discharge heavy metal-containing effluents into nearby water bodies, posing serious threats to aquatic flora and fauna [[1], [2], [3], [4]]. Several studies have been conducted to detect heavy metals in water and sediments, of polluted aquatic environments of Bangladesh which included the Turag River ([1,5,6]; Arefin et al., 2016 [7], [[8], [9], [10]]), the Shitalakhya River [1,11], Buriganga River [1,5,6,12] different lakes in Dhaka city [13], the Karatoya River [[14], [15], [16]], wetlands near the Barapukuria coal mining zone of Dinajpur district [[17], [18], [19], [20], [21]], Atrai River [22], Dharla river [23], the Old Bhramaputra River [24], and Chalan *Beel* wetland [25].

The Turag River, which flows alongside Bangladesh's capital city, Dhaka, is the country's most polluted river, receiving a variety of heavy metals discharged from industrial wastewater [[6], [8], [9], [10],^12^,^26^]. On the other hand, urban rivers such as Karatoya, located in the northwestern part of the country, are also critically polluted [14,27]. Researchers have detected the pollution of wetlands and rivers in the northwest regions of Bangladesh from the effluents of coal mining industries and thermal power plants [[17], [18], [19], [20]]. By releasing heavy and non-heavy toxic metal compounds into the air and water, elevated concentrations of heavy metals (HM) and toxic substances result in the contamination of water sources, which affects areas beyond the immediate vicinity of the mine, such as the freshwater habitat Ashura *Beel* of Dinajpur, Bangladesh [17,19,21]. Compared to the central and southern regions of Bangladesh, less industrial development occurred in the northwestern part of the country; therefore, rivers such as the Atrai and Dhepa are assumed to be less polluted and considered indigenous species-rich fish habitats in the northwest region of Bangladesh [22,28,29].

Heavy metal concentrations above threshold levels cause bioaccumulation in fishes and other aquatic organisms of both freshwater, coastal and marine ecosystems, which have been detected globally ([15,[30], [31], [32], [33], [34], [35], [36], [37]]; [38]). Many authors have detected the impacts of heavy metal bioaccumulation on fish's lives in different countries. The studies included both laboratory-exposed experimentation and wild-collected specimen analyses, demonstrated that heavy metal pollution in aquatic environments caused growth retardation and size variation ([39]; Naz et al., 2022 [35,[40], [41], [42]]; Canli., & Atli, 2003), biochemical and histopahtological changes ([35,43,44]; [45]; [46]), and reproductive disturbances as oestrogenic, anti-oestrogenic, thyroid disrupting, androgenic, or anti-androgenic effects impaired processes of the endocrine system associated with reproduction ([[47], [48], [49], [50]]; Abdel-Kader & Mourad, 2019 [51]; [52]; Alquezar et al., 2016 [53,54]; [55]; [56]).

Researchers reported heavy metal bioaccumulation in several fishes and the associated human health risks from different water bodies in Bangladesh. Most of the studies were from Dhaka-centered Rivers such as the Turag, Buriganga, and Dhaleswari that used a wide range of indigenous fish species such as *Channa punctata, C. striata, Mastacembelus armatus*, *Glossogobius giuris*, *Amblypharyngodon microlepis*, *Heteropneustes fossilis, Puntius ticto, P. sophore, P. chola, Labeo rohita* ([[57], [58], [59]]; [60]; [5,8,61,62]). Few studies detected heavy metal bioaccumulation in fishes and determined the human health risk in rivers of the country's other regions, such as Karnofully River in the south [63], Old Brahmaputra River in the middle part of the country [64]; Padma River in the northwest part of the country [65], Karatoya River in the northern part of the country [14]. All the studies concentrated on identifying heavy metal bioaccumulation and the associated human health risks. Heavy metal detection was not found in fish from the Atrai River, Dharla River, and Ashura *Beel* located in the northwest regions of Bangladesh. Moreover, we did not find studies demonstrating the impacts of heavy metal pollution on aquatic life in polluted freshwater environments or laboratory-exposed experimentation in Bangladesh. Therefore, this study aimed to detect heavy metal bioaccumulation in fishes and to evaluate the hypothesis that heavy metal contamination may impact the growth and reproduction of aquatic organisms living in freshwater ecosystems. The study monitored the growth and reproductive health of wild-caught *Channa punctata* [66] from five freshwater habitats, viz, the Atrai River, Ashura *Beel*, Dharla River, Karatoya River, and Turag River. The investigation detected heavy metal bioaccumulation in muscle tissues of wild-caught *C. punctata*, estimated the length-weight relationship and condition factor for its growth pattern and health status, and determined size at first sexual maturity, ova diameter, fecundity, and gonado-somatic index for its reproductive health. Finally, the relationship of the heavy metal bioaccumulation with the growth and reproduction of *C. punctata* was determined using Pearson correlation analyses. *We selected the* spotted snakehead, *Channa punctata* [66], *as an experimental fish due to its availability in Bangladesh's freshwater ecosystems throughout the year* [67] and as well as its *high carnivorous and predatory nature, which allows it to absorb high concentrations of toxins from the surrounding environment* [68,69]*.* Moreover, its hardy nature and tolerance to poorly oxygenated water make it a suitable fish species for monitoring of aquatic pollution [33,69]. Previous studies have reported the use of *C*. *punctata* in a diverse range of experiments related to heavy metal exposure and its effects on fish's lives, including biomarkers of oxidative stress, associated genotoxicity, and histopathology [46,70,55].

## 2 Materials and methods

### 2.1 Habitats and sample collection sites selection

**Fig. 1:**
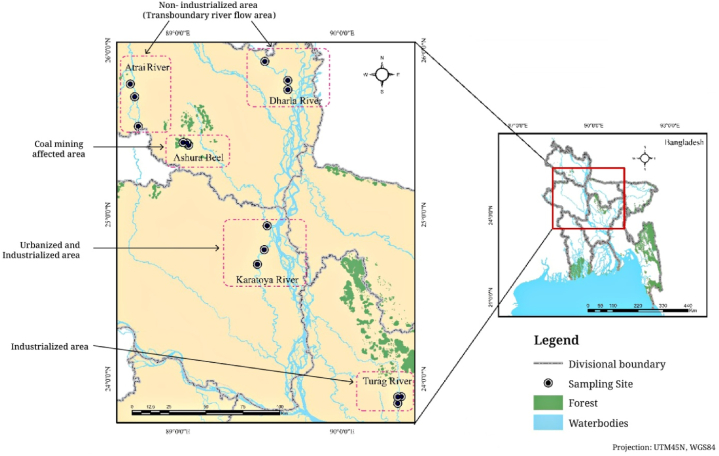
The map displays sample collection sites from five freshwater habitats in Bangladesh. The Turag River represents the industrialized area; the Karatoya River represents the urban and industrialized area; the Ashura *Beel* represents the coal mining areas; the Atrai and Dharla Rivers represent the non-industrialized areas and Transboundary Rivers.

The study selected five freshwater habitats from the middle and northwestern regions of Bangladesh (Fig. 1). The five habitats were the Turag River (considered a heavily polluted industrialized area) located in Gazipur district, the Karatoya River (considered an urbanized and industrialized area) located in Bogura district, Ashura *Beel*, a natural depression/wetland (considered as a coal mining affected area) located in Dinajpur district, the Atrai River, a transboundary river that connects India and Bangladesh, located in Dinajpur district (a non-industrialized area), and the Dharla River (considered a non-industrialized area) located in Kurigram district.

### 2.2 Fish sample collection and transprotaton

From March to October 2023, we collected a total of 30 fish samples from fishermen's catch of each habitat. During this period, the gonadal development and spawning activities of freshwater fish in Bangladesh mostly occur. Local fishermen collected the fish samples using traditional fishing gear, which included a cast net, seine net, samples, and lift net. We transported the live fish samples from the Atrai River and Ashura *Beel* to the Fisheries Biology and Genetics laboratory of Hajee Mohammad Danesh Science and Technology University (HSTU), while we transported the frozen fish samples from distantly located habitats such as the Dharla River, Karatoya River, and Turag River for data collection.

### 2.3 Identification of female *C. punctata* and measurements

We collected data on ova diameter, fecundity, and size at first sexual maturity from only the female specimen among the collected fish samples. The current study evaluated a total of 800 fish specimens, identifying 436 females and 364 males with a ratio of 1.09:0.91. Month- and habitat-wise sex ratios of collected *C. punctata* from 5 habitats showed similar ratios (data not display). We measured the body weight (BW) and standard length (SL) of the collected samples to the nearest 0.01 g and 0.1 cm, respectively.

### 2.4 Detection of heavy metal bioaccumulation in the collected fish

We selected ten *C. punctata* individuals from each habitat's collected specimens in June and dissected each to obtain muscle samples. We followed a slightly modified version of Atta et al.'s (2012) protocol for the digestion process. June marked the month when prominent spawning activities of *C. punctata* took place*.* To detect heavy metal bioaccumulation, we selected samples from this month. We conducted atomic absorption spectroscopy analysis using a flame atomic absorption spectrometer (Model: AA240FS, Varian, Australia). In summary, we used a heated plate in a fume hood to digest 1 g of the materials in 5 mL of concentrated HNO3. After digestion, we cooled the samples and filled a volumetric flask to 100 mL. Next, we filtered the sample (125 mm dia.∗100 circles) using Whatman TM qualitative 1-μm filter paper and analyzed it in a flame atomic absorption spectrometer (Model: AA240FS, Varian, Australia). We prepared calibration standards by diluting 1000 mg/L stock metal standard solutions (Fluka Analytical, Sigma-Aldrich, Germany) during the analysis to measure selected metals such as Cd, Cr, Hg, and Pb. We used the mean result of three successive replicates for each measurement. We selected the heavy metals based on the pollution levels in the selected sites. Pb, Cd, Cr, Cu, Hg, and Fe were the heavy metals detected in the Turag River, Karatoya River, and Ashura *Beel*, while Pb, Cd, and Cr were common in most areas. In review, Islam et al. [1] identified the Turag River, Karatoya River and the coal mining zone (Ashura *Beel),* the hotspot of Pb, Cd and Cr in Bangladesh. Therefore, in this study, we selected Pb, Cd, and Cr as common in all the locations, and Hg was selected by considering the use of battery-driven vehicles in the urban and rural areas of Bangladesh that may contaminate the nearby waterbodies.

### 2.5 Interpretation of growth of *C. punctata* through length-weight relationship and Fulton's condition factor (F) analysis

After log-transformation, we used the equation W = a $L^{b\;}$ (1) to estimate the length-weight relationship: log (W) = log (a) + *b* log (L) (2). We calculated the parameters *a* (intercept) and b (the slope of the relationship) using linear regression. The ideal value of *b* = 3.0 represents isometric growth, and any deviation from that value is considered an allometric growth pattern [71]. The condition factor (K) describes the ratio between fish body weight and length, reflecting the interactions between biotic and abiotic variables in the fish's physiological condition [72]. We calculated the Fulton's condition factor K using Fulton's (1904) equation, K = 100∗ (W/ $L^{3})$ (3.0), where W represents the body weight (BW) and L represents the total length (TL). The condition factor (K) > 1.0 indicates the fish's well-being in an environment.

### **2.6** Reproductive biology of *C*. *punctata* collected from five habitats

#### 2.6.1 Determination of size at first sexual maturity (L_m_)

The empirical model log (*Lm)* = −0.1189 + 0.9157∗ log (*L*_max_) (4), according to Binohlan and Froese [73], was used to estimate the size at first sexual maturity of *C. punctata*. We conducted the analysis of L50 by plotting the percentage of mature individuals (PMI) against total length (TL) to create a logistic curve [74].

#### 2.6.2 Determination of ova diameter

We cut small pieces of ovaries from the anterior, middle, and posterior regions, then teased out the ova onto a glass slide. We measured a total of 300 ova in mm from each individual fish at various stages of maturity to estimate the mean oocyte diameter per ovarian stage [75]. Ova were selected on a random basis and ova diameter was measured by a camera microscope attached to a micrometer.

#### 2.6.3 Estimation of gonadosomatic index (GSI)

We randomly selected 50 female fish (10 from each water body) each month to estimate the gonadosomatic index (GSI). We measured each fish's total weight (BW) to the nearest (0.01) gram. We disassembled the fish to remove the entire gonad, and then used an electronic balance to measure their weight (GW) to the nearest 0.001 g before preserving them in 5 % formalin for further research. To estimate the GSI, the formula GSI = (GW ÷ BW) × 100 (5) was used.

#### 2.6.4 Fecundity

Following a macroscopical examination, the gonadal stages of *Channa punctata* were categorized in accordance with Nikolskii [76] and are as follows: Immature is Stage I; maturing is Stage II; developed is Stage III; ripe is Stage IV; and spent is Stage V. We only considered the ovaries of the mature (Stage III) and ripe (Stage IV) specimens when estimating fecundity. We determined fecundity using the sub-sampling method [77,78]. Only the mature ova at stages III and IV were selected for the fecundity estimation. Gravimetric methods, as described by Murua et al. [79], determined the monthly estimation of fecundity for each stock. We selected approximately 50 % of mature females at stages III and IV of oocyte maturation from monthly samples. We took three 0.1–0.2 g sub-samples of ovaries from the anterior, posterior, and middle of the gonad and immersed them in a solution containing 60 mL of ethanol, 30 mL of formaldehyde, and 10 mL of glacial acetic acid. We used the solution to wash the mucus and prevent the eggs from sticking together, facilitating observation. We counted the eggs using a stereo light microscope (Nikon, YS-100) after placing them in a dish. The gravimetric method determined the relative fecundity by taking the mean of the three subsamples and applying the following formula.

Fe = (N × gonad weight) ÷ weight of sub-sample (6), where, N = number of mature eggs (maturation stages III and IV) and Fe = fecundity.

### *2.7* Statistical analyses

We used the Shapiro-Wilk test [80] to test the normality of the original data, which included body weight, length, GSI, ova diameter, fecundity, cadmium, chromium, mercury, and lead, before proceeding with the analyses. We found that all the data, except for the heavy metal concentration, had a normal distribution. Therefore, we transformed only the heavy metal data using log10 (variables+1) prior to analysis [81]. We also tested the homogeneity of the data's variances. We analyzed the means using a one-way ANOVA, ensuring a significance level of less than 0.05. We performed multiple comparison procedures using the multicomp [82] R package. The Tukey's post hoc test due to its conservative approach in separating treatment means was employed. We used a pairwise *t*-test to compare group levels pairwise, incorporating corrections for multiple testing. We used the sizeMat R [83] package to determine the length at which 50 % of fish reached sexual maturity (L50), based on the ratio of the coefficients of a binary logistic regression of length and maturity level (mature; immature individuals). We performed the Pearson correlation analysis in R-Studio (version 2023.09), utilizing the AgroR package [84] for calculations, the Tidyverse [85] R package for data processing and plotting, and the ggpubr R package for simple ggplot2-based data visualization [86]. Pearson correlation analysis evaluates the strength and direction of the linear relationship between two variables, represented by the correlation coefficient, R, which ranges from −1 to +1. An R-value of +1 signifies a perfect positive linear relationship, while an R-value of −1 indicates a perfect negative linear relationship. An R-value of 0 suggests no linear relationship (Akoğlu, 2018). Furthermore, an R-value of ≥0.7 indicates a strong correlation, an R-value of ≥0.5 denotes a weak correlation, and a value in the range of 0.5 ≤ |R| < 0.7 represents a moderate correlation [87]. Pearson correlation is particularly suitable for analyzing continuous, normally distributed data, which characterizes the variables examined in our study, such as ova diameter, fecundity, and the gonadal-somatic index (GSI).

## 3 Results

### **3.1** Heavy metal bioaccumulation in *C. punctata*

The concentrations of all the heavy metals except mercury were highest in the Turag River and lowest in the Dharla River stock of *C. punctata* (Table 1). The concentrations of Cd were 0.240 ± 0.007 mg/kg, 0.217 ± 0.004 mg/kg, 0.197 ± 0.004 mg/kg, 0.103 ± 0.004 and 0.097 ± 0.004 mg/kg were found in *C. punctata* of the Turag, Karatoya, Aushura, Atrai and Dharla River, respectively (Table 1). A similar hierarchy in Cr concentration level was found in *C. punctata* of Turag, Karatoya, Aushura, Atrai, and Dharla River, which were 0.407 ± 0.011 mg/kg, 0.283 ± 0.020 mg/kg, 0.193 ± 0.011 mg/kg, 0.017 ± 0.004 mg/kg, and 0.011 ± 0.001 mg/kg, respectively. Similarly, Cd and Cr, the highest level of Pb concentration was found in the *C. punctata* of Turag River (1.697 0.022 mg/kg), followed by Ashura *Beel* (1.397 0.015 mg/kg), Karatoya River (1.153 0.015 mg/kg), Atrai River (0.663 0.015 mg/kg), and Dharla River (0.527 0.015 mg/kg). Mercury (Hg) concentrations were relatively lower in the *C. punctata* in all the habitats, with the highest in the Karatoya River (0.093 ± 0.004 mg/kg) followed by the Turag River (0.033 ± 0.004 mg/kg), Ashura *Beel* (0.030 ± 0.007 mg/kg), Atrai River (0.023 ± 0.004 mg/kg) and Dharla River (0.012 ± 0.002 mg/kg). According to FAO/WHO 2003 [88], only the concentrations of Mercury in all the habitats were below (<0.5) the maximum allowable concentration (MAC), and the Chromium concentrations were found below (<0.15) the MAC in Dhalra and Atrai River (Table 1). On the other hand, the concentrations of Cadmium (>0.05) and Lead (>0.5) were above the MAC level in all the studied habitats, indicating human health risks upon consumption of fish of these habitats. The heavy metals studied were found in the following order: Pb > Cd > Cr > Hg in Ashura *Beel*, Pb > Cd > Cr > Hg in the Atrai River, Pb > Cd > Cr > Hg in the Dharla River, Pb > Cd > Hg > Cr in the Karatoya River, and Pb > Cr > Cd > Hg in the Turag River (Table 1).

**Table 1:** Bioaccumulation of heavy metals in muscles of *C. punctata* (mean ± SE).

Metals studied	Heavy metal Concentration (mg/kg/wet-wt)					
	Ashura *Beel*	Atrai River	Dharla River	Karatoya River	Turag River	MAC [88]
Cadmium (Cd)	0.197 ± 0.004	0.103 ± 0.004	0.097 ± 0.004	0.217 ± 0.004	0.240 ± 0.007	0.05
Chromium (Cr)	0.193 ± 0.011	0.017 ± 0.004	0.011 ± 0.001	0.283 ± 0.020	0.407 ± 0.011	0.15
Mercury (Hg)	0.030 ± 0.007	0.023 ± 0.004	0.012 ± 0.002	0.093 ± 0.004	0.033 ± 0.004	0.5
Lead (Pb)	1.397 ± 0.015	0.663 ± 0.015	0.527 ± 0.015	1.153 ± 0.011	1.697 ± 0.022	0. 5

### 3.2 Growth pattern of the *C. punctata*

The growth parameter (*b*) obtained from LWRs values varied from stock to stock with a range of 2.405–3.051, indicating the negative allometric growth pattern in all the stocks except Dharla river stock (Table 2). The Dharla River had the highest b value (b = 3.051), nearly matching the isometric growth pattern (b = 3.0). The Fulton condition factors of three habitats, Ashura *Beel*, Atrai River, and Dharla River, were in good health (K > 1.0), while *C. punctata* of Turag and Karatoya River stock showed poor health (K < 1.0) (Table 2)

**Table 2:** Length-weight relationship (slope, *b*) and condition factors (F) of *Channa punctata* collected from the selected freshwater fish habitat of Bangladesh.

Habitat	Sample number (N)	Slope, *b*	Growth Pattern interface	Condition Factor (F)	Health status
Ashura *Beel*	50	2.943	Negative Allometric	1.056	Good
Atrai River	50	2.945	Negative Allometric	1.102	Good
Dharla River	50	3.051	Positive Allometric	1.208	Good
Karatoya River	50	2.846	Negative Allometric	0.946	Poor
Turag River	36	2.405	Negative Allometric	0.896	Poor

### 3.3 Reproductive biology of *C*. *punctata* collected from different habitats

**Fig. 2:**
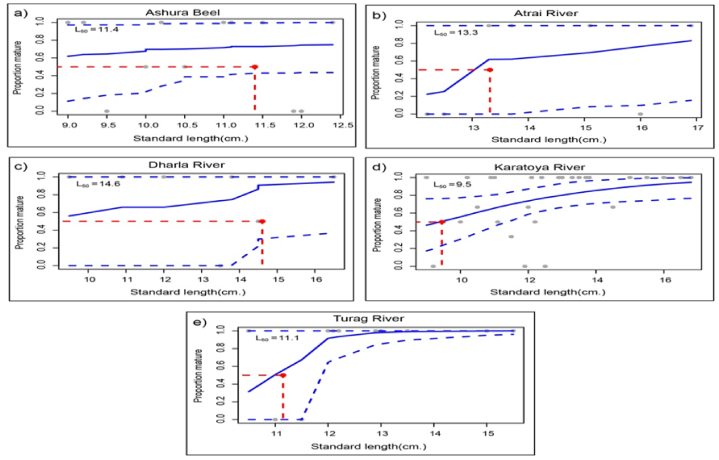
Length at first sexual maturity of *C. punctata* from five different stocks.

We estimated the length at first sexual maturity (L50) using the derived curves from logistic regression analyses of each habitat. Fig. 2(a–e) plots the proportion of mature individuals on the ordinate and standard length for C. punctatus in each habitat. The Dharla River stock (Fig. 2c) exhibited the highest L50 value of 14.6 cm, with the stocks from Atrai River (Fig. 2b), Ashura Beel (Fig. 2a), Turag River (Fig. 2e), and Karatoya River (Fig. 2d) following at 13.3, 11.4, 11.1, and 9.5 cm, respectively. The data suggest significant differences in size at first sexual maturity among the fish stocks of five habitats.

The ova diameter of all the stocks of *C. punctata* showed a generally increasing trend from March to July, followed by a decrease in August to October (Table 3). All the stocks showed the highest ova diameter in the month of July, ranging from the lowest (0.54 ± 0.38 mm) in the Turag River stocks to the highest (1.01 ± 0.71 mm) in the Dharla River stocks (Table 3).

**Table 3:** Stock-wise variation of ova diameter (mm) of *C. punctata* in different months.

Habitats	March	April	May	June	July	August	September	October
Ashura	0.30 ± 0.01	0.38 ± 0.01	0.35 ± 0.02	0.77 ± 0.01	0.83 ± 0.01	0.72 ± 0.02	0.55 ± 0.02	0.22 ± 0.03
Atrai	0.35 ± 0.01	0.43 ± 0.03	0.49 ± 0.01	0.85 ± 0.03	0.96 ± 0.02	0.82 ± 0.03	0.68 ± 0.02	0.30 ± 0.01
Dharla	0.35 ± 0.25	0.47 ± 0.33	0.58 ± 0.41	0.91 ± 0.64	1.01 ± 0.71	0.91 ± 0.64	0.70 ± 0.49	0.31 ± 0.22
Karatoya	0.24 ± 0.01	0.36 ± 0.00	0.31 ± 0.01	0.63 ± 0.02	0.73 ± 0.01	0.67 ± 0.01	0.43 ± 0.02	0.18 ± 0.01
Turag	0.16 ± 0.11	0.22 ± 0.15	0.28 ± 0.19	0.48 ± 0.33	0.54 ± 0.38	0.39 ± 0.27	0.30 ± 0.21	0.12 ± 0.08

The fecundity illustrates a clear seasonal pattern, with all stocks showing an increasing trend from March with a peak in July and then declining towards October (Table 4). The highest fecundity was found in Dharla stocks (25276 ± 912) in June, which indicates that this stock might have the best reproductive capacity due to favorable environmental conditions. In contrast, the Turag River with the lowest peak (12289.33 ± 2410.54) in the same month might represent stocks under suboptimal conditions.

**Table 4:** Stock-wise variation of the fecundity of *Channa punctata* in different months.

	March	April	May	June	July	August	September	October
Ashura	4093 ± 322	5255 ± 362	8470 ± 509	12238 ± 840	16712 ± 696	13036 ± 897	6286 ± 441	3211 ± 604
Dharla	6873 ± 829	15430 ± 970	19864 ± 939	25276 ± 912	24880 ± 739	18678 ± 972	11177 ± 842	7711 ± 661
Atrai	5291 ± 700	13131 ± 102	16444 ± 514	19706 ± 944	22745 ± 977	18444 ± 991	9401 ± 997	6198 ± 680
Karatoyaa	4328 ± 481	4452 ± 413	7431 ± 996	12769 ± 834	16536 ± 919	10550.±938	6715 ± 856	2851 ± 211
Turag	3276 ± 538	3745 ± 580	6031 ± 908	10661 ± 980	12289 ± 987	8871 ± 796	5788 ± 626	2711 ± 256

This study examines the temporal variation in the Gonado-somatic Index (GSI) of various stocks, providing insights into the reproductive cycles and potential environmental influences on *C. punctata* (Table 5*)*. The highest GSI was observed in Dharla stocks (8.20 ± 0.78) in July with a longer duration of high reproductive activity, ranging from May (7.19 ± 0.78) to August (7.18 ± 0.48). The Atrai River stock showed moderate GSI values with a peak in July (7.89 ± 0.65) and displayed a long duration of reproductive activity ranging from May (6.88 ± 0.22) to July (7.89 ± 0.65). The other three stocks showed poor GSI values, in which the peak season of GSI ranged from 4.18 ± 0.69 in May and 4.61 ± 0.46 in August, with the highest value of 6.39 in July for the Karatoya River stock (Table 5).

**Table 5:** GSI variation of five different fish stocks (mean ± SE).

	March	April	May	June	July	August	September	October
Ashura	2.53 ± 0.34	3.08 ± 0.19	5.18 ± 0.23	6.07 ± 0.45	6.05 ± 0.51	4.43 ± 0.37	3.92 ± 0.54	2.44 ± 0.35
Atrai	2.69 ± 0.35	3.25 ± 0.32	6.88 ± 0.22	7.20 ± 0.56	7.89 ± 0.65	5.79 ± 0.46	4.13 ± 0.61	2.38 ± 0.39
Dharla	2.79 ± 0.64	3.89 ± 0.24	7.19 ± 0.78	7.69 ± 0.60	8.20 ± 0.78	7.18 ± 0.48	4.77 ± 0.61	2.11 ± 0.24
Kartoya	2.46 ± 0.40	3.42 ± 0.53	4.46 ± 0.46	5.76 ± 1.11	6.39 ± 0.99	4.11 ± 0.73	3.99 ± 0.63	2.64 ± 0.33
Turag	2.11 ± 0.21	3.11 ± 0.63	4.18 ± 0.69	4.67 ± 0.67	5.03 ± 0.70	4.61 ± 0.46	3.95 ± 0.40	2.57 ± 0.41

### 3.4 Determination of heavy metal pollution and reproductive health of *C. punctata*

**Fig. 3:**
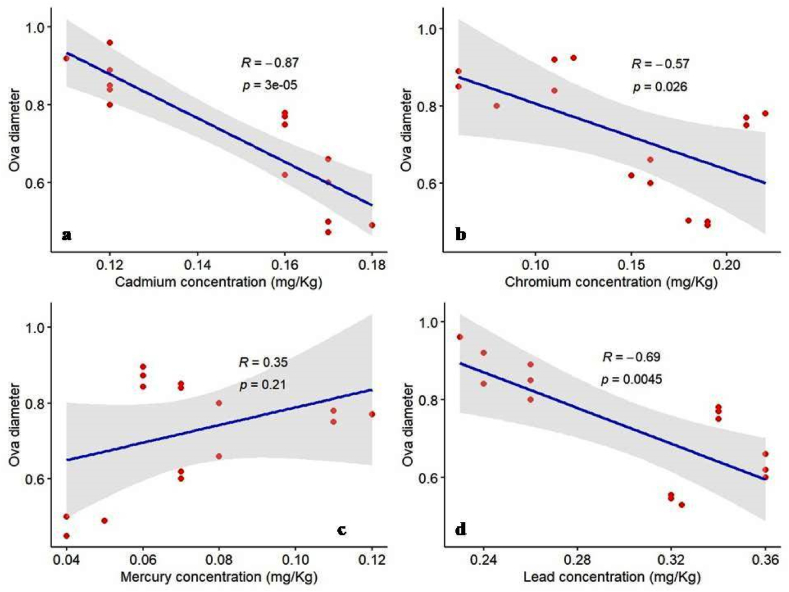
Pearson correlation between ova diameter and four heavy metals extracted from muscle tissue of *C. punctata*, the relationship of: a) ova diameter and cadmium concentration b) ova diameter and chromium concentration c) ova diameter and mercury concentration and d) ova diameter and lead concentration.

We depicted the impacts of heavy metal bioaccumulation on the reproductive performance of *C*. *punctata* in wild stocks using Pearson correlation coefficient analyses. Observing significant variations in bioaccumulation of heavy metal concentration and reproductive traits across different stocks of *C*. *punctata*, we conducted a Pearson correlation coefficient analysis to test the relationship between the level of heavy metal bioaccumulation and reproductive performance. A statistically significant, strong negative correlation indicates a small R-value and low p-value. The R-values of cadmium vs. ova diameter, chromium vs. ova diameter, and lead vs. ova diameter were small, indeed negative, and the p-values were very low (Fig. 3: a, b, d). Therefore, the concentrations of cadmium, chromium, and lead strongly suggest that higher values of these metals are associated with smaller ova diameter (Fig. 3: a, b, d). The relationship revealed no reliable evidence that mercury concentration affects ova diameter in the studied area, which was indicative of a positive R value (0.35) and a higher p-value (0.21) (Fig. 3 c).

**Fig. 4:**
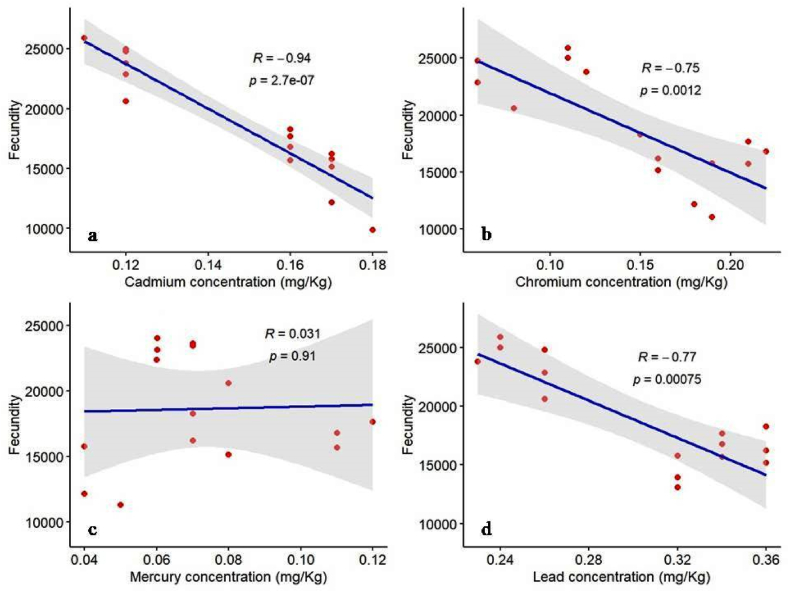
Pearson correlation between fecundity and four heavy metals extracted from muscle tissue of *C. punctata*, the relationship of: a) fecundity and cadmium concentration b) fecundity and chromium concentration c) fecundity and mercury concentration and d) fecundity and lead concentration.

Cadmium (R: 0.94; p-value:2.7e-07) had the strongest negative impact on fecundity, followed by chromium (R: 0.75; p-value:0.0012) and lead (R: 0.77; p-value:0.00075), mercury showed no significant correlation (R:0.031; p-value:0.91) (Fig. 4:a-d). These results could indicate the toxic effects of certain heavy metals on reproductive health.

**Fig. 5:**
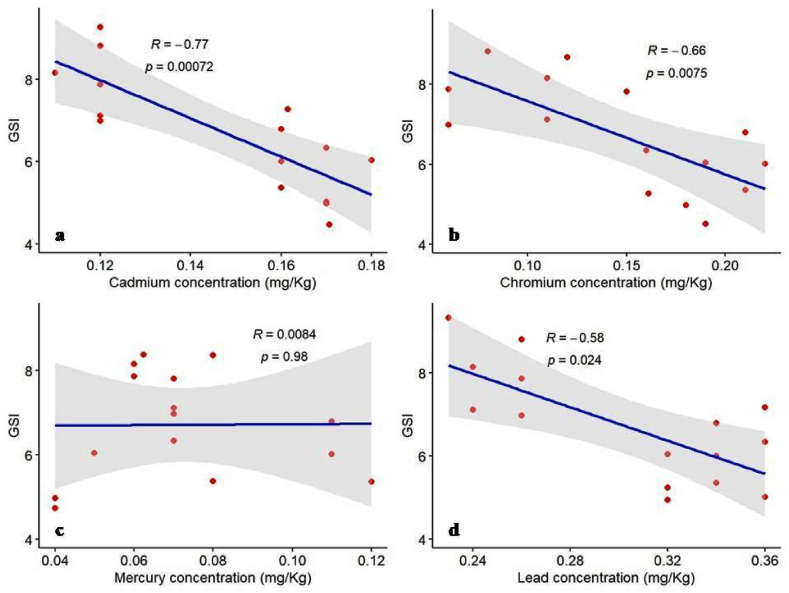
Pearson correlation between GSI and four heavy metals extracted from muscle tissue of *C. punctata*, the relationship of: a) GSI and cadmium concentration; b) GSI and chromium concentration; c) GSI and mercury concentration; and d) GSI and lead concentration.

The impact of heavy metal bioaccumulation on the gonado-somatic index (GSI) depicted a significant inverse relationship with cadmium, chromium, and lead concentration (Fig. 5:a,b,d). In constrast, mercury concentration did not show such a relationship (Fig. 5:c). The correlation analysis across the four heavy metals indicated that cadmium (R: 0.77, p-value: 0.00072) and chromium (R: 0.66, p-value: 0.0075) substantially negatively impact GSI. At the same time, lead shows a moderate negative impact (R: 0.58, p-value:0.024), and mercury shows no significant impact (R:0.0084, p-value:0.98). This suggests a robust inverse relationship, where higher cadmium, chromium, and lead concentrations are associated with a significant decrease in GSI.

## 4 Discussion

### 4.1 Heavy metal bioaccumulation in *C. punctata*

Muscle samples of *C. punctata* from all habitats showed bioaccumulation of Cd, Cr, Hg, and Pb, with varying concentration levels. The levels of bioaccumulation of Cd and Pb in fishes at all the sites were higher than the maximum acceptable limit recommended by FAO/WHO [88]. The Cr concentration was higher than the MAC in *C. punctata* Turag River, Karatoya River, and Aushura Beel and lower in *C. punctata* of Atrai River and Dharla River. We detected the Hg concentration in the *C. punctata* of the studied habitats for the first time in all locations. However, its concentration was lower than the acceptable 0.5 mg/kg [88]. Several authors have previously reported the detection of heavy metals in fish of different water bodies from Bangladesh [11,13,16,21,24,[89], [90], [91]]. Assimilation and bioaccumulation of Cd and Cr occurred in a wide range of fish species, including *Channa punctata* [8], *Cyprinus carpio* [92], three carp species [34], and *Cirrhinus mrigala* [93]. Several studies have previously detected bioaccumulation of lead (Pb) in different organs of fish (Hosain et al., 2021; [[94], [95], [96], [97]]). Mercury (Hg) concentrations were relatively lower in the *C. punctata* in all the habitats of this study, with the highest in the Karatoya River stock (0.093 ± 0.004 mg/kg) and lowest in the Dharla River stock (0.012 ± 0.002 mg/kg). Mercury (Hg) concentrations were relatively lower in the *C. punctata* in all the habitats of this study, with the highest in the Karatoya River stock (0.093 ± 0.004 mg/kg) and lowest in the Dharla River stock (0.012 ± 0.002 mg/kg). Afrin et al. [9] reported that they detected mercury in fishes from Turag in very few cases among the studied habitats, and in most cases, they could not detect Hg values. Minimal (Hg values ranging from 0.00012 to 0.00145 ppm) or no Hg pollution were detected by Rahman et al. [26] at Ashulia point in the Turag River. In contrast, Khan et al. (2007) did not detect Hg at Ashulia point in the Turag River. Previous studies did not detect Hg in Ashura, Atrai, Dharla, and Karatoya Rivers fish. However, the mercury concentrations in *Channa punctata*, *Mastacembelus armatus*, *Mystus vittatus*, *Puntius puntio*, and *Amblyceps mangois* were detected from the Dhaleswari River, which ranged from 0.004 to 0.011 mg/kg [61,98].

Among the habitats investigated, the Turag River was most polluted by heavy metals, as the concentrations of three metals, such as Cd, Cr and Pb, was highest, followed by the Karatoya River, where the concentration of Hg was highest and Cd and Cr were just after the level found in the Turag River. Ashura *Beel* may be considered moderately polluted because all of the heavy metal concentrations were below the Turag and Karatoya Rivers but above the Atrai and Dharla Rivers. On the other hand, the Dharla River was least polluted by heavy metal pollutants, as the concentration of the metals was lowest. Based on the findings, we can conclude that the Turag River has the highest pollution levels and Dharla River has the lowest pollution levels among the studied 5 freshwater habitats of Bangladesh. Other studies supported our findings as they identified the Turag River as the most polluted river in the country due to the discharge of a variety of heavy metals from industrial wastewater [6,99,100]. Moreover, it is evident that the majority of consumer goods industries discharge their effluents into the Turag River either directly or indirectly without any treatment, which pollutes the surface water ([12,101]; Arefin et al., 2016 [7]; [[8], [9], [10],^26^]). Industrial discharges significantly pollute the Karatoya River, one of the urban areas' rivers [14,27]. Coal mining and thermal power plant operations contribute to environmental pollution by releasing heavy and non-heavy toxic metal compounds into the air and water. These elevated concentrations of heavy metals (HM) and toxic substances contaminate water sources, affecting areas beyond the immediate vicinity of the mine, such as the freshwater habitat Ashura Beel in Dinajpur, Bangladesh [[17], [18], [19], [20], [21]]. Sayem et al., 's 2023 study found the Atrai to have a lower pollution level, potentially due to non-industrialized areas. The lowest pollution level in the Dharla River could potentially be attributed to the absence of significant industrial development in the nearby city of Kurigram, located in the Rangpur division of Bangladesh. These two transboundary rivers' lower heavy metal pollution levels also indicate that there is no or very little industrial discharge in the Indian part of the rivers.

### 4.2 Growth pattern of the *C. punctata*

We interpreted the growth patterns of the studied stocks of *C*. *punctata* using the length-weight relationship (LWR) and Fulton's condition factor (F) values. We used the slope (b) from LWR to determine the fish growth pattern. We used the fish body condition factor (F) to confirm any variation in fish well-being among the habitats. The growth parameter and the Fulton condition factors indicates the negative allometric growth pattern and poor wellness status *C. punctatus* of Turag River, Karatoya River and Ashura *Beel* where the level of heavy metal bioaccumulation were more than two other habitats. On the other hand, positive allometric growth pattern and wellness of health status were found in the Dharla river stock where the concentration of heavy metal bioaccumulation in *C. punctatus* were least. The negative allometric growth patterns and poor wellness status of the stocks may be due to a lack of natural feed, heavy metal water pollution, poor genetic quality, and so on. Studies on wild fish revealed that bioaccumulation of heavy metal concentration and fish size were found to be negatively correlated by several authors [41,42,102,103]. The relationships between the concentrations of heavy metals in fish muscles and the condition factor of fish samples showed opposing trends [103]. Laboratory-induced studies may confirm the phenomenon, as they report a negative relationship between heavy metal bioaccumulation and fish growth and survival. The presence of Cd reduced the growth and survival in *Mystus seenghala* [104] and in juvenile channel catfish, *Ictalurus punctata* [40]. Exposure to Cr and Cd significantly reduced the weight gain of *Channa marulius* [105]. When exposed to four different concentrations of waterborne Cr, *Platichthys stellatus* showed decreases in daily weight gain, condition factor, and daily length gain [43]. Abdel Hakim et al. (2016) [106] observed significant reductions in the specific growth rate (SGR), weight gain (WG), feed conversion ratio (FCR), and survival rate of Nile tilapia (Oreochromis niloticus) under sub-lethal exposure of Cu, Hg, Pb, and Cd. The mixture of heavy metals exposed to fish showed a significant reduction in wet weight gain, fork length, and total length [44,107,108].

### 4.3 Reproductive biology of *C*. *punctata* collected from different habitats

During the study period, we estimated the length at first sexual maturity, ova diameter, fecundity, and gonadosomatic index (GSI) to reveal the reproductive status of the *C. punctatus* of studied habitats. The data suggest significant differences in size at first sexual maturity among the fish stocks of five habitats. The results obtained in our study are consistent with Prasad et al. [109] and Hasan et al. [90], who reported that the length at first sexual maturity of *C*. *punctata* was 12.0 cm in the Guntur River, India, 12.5 cm in the Varuna River, India, and 11.65 cm from Gajner Beel, Bangladesh. According to this study, the highest L50 in the Dharla River stock suggests that the fish mature at a significantly larger size compared to the *C*. *punctata* of other habitats, which reflects a higher growth rate which could possibly influence differing environmental conditions or genetic factors. The highest ova diameter of all *C. punctatus* stocks were found in the month of July. Among the stocks, the highest ova diameter (1.01 ± 0.71 mm) was found in the Dharla River stocks. In July, the largest mean ova diameter of *C*. *punctata* was 1.01 ± 0.71 mm, which was higher than the previous study reported by Ali [110], similar to the study of Narejo et al. [111]. The Dharla stock consistently displays larger ova diameters than other stocks, particularly from June to August, which may be due to the abundance of natural feed and less river pollution. The fecundity indicated the seasonal pattern, with an increasing trend from March with a peak in July and then declining towards October for all the stocks. This pattern suggests that the reproductive peak for *C. punctata* occurs in mid-summer, which is consistent with the species' breeding habits in response to monsoon rains and temperature changes in their natural habitats. Fish fecundity typically varies within the same population and is species-specific, potentially due to various factors like age, size, body and gonad weight, and the ecological conditions of the water body, among others [112]. The highest fecundity was found in Dharla stocks which indicate that this stock might have the best reproductive capacity due to favorable environmental conditions. In contrast, the Turag River with the lowest peak in the same month might represent stocks under suboptimal conditions. This study examines the temporal variation in the Gonado-somatic Index (GSI) of various stocks, providing insights into the reproductive cycles and potential environmental influences on *C. punctata*. The highest GSI was observed in Dharla stocks (in July with a longer duration of high reproductive activity, ranging from May (7.19 ± 0.78) to August (7.18 ± 0.48). Several authors reported the maximum GSI value of *C*. *punctata* during June–July, with a maximum of mature individuals, and the lowest GSI from November to February [20,109].

### 4.4 Relationship between heavy metal pollution and the reproductive performance of *C. punctata*

Several authors have previously reported that heavy metal contamination affects reproductive activity of fish through laboratory trials of heavy metal-exposed fish as well as in wild-collected fish. The significant reduction of GSI, fecundity, fertilization, and hatching rate in heavy metal-accumulated fish were reported [49,54,113,114]. Researchers have found that heavy metals interfere with gametogenesis by influencing egg quality and fecundity, as well as the endocrine system, hatching, and larval development [50,52,115,116]. Cadmium and chromium showed the strongest negative impact on fecundity, ova diameter, and GSI, followed by lead, while mercury showed no significant correlation. The chronic exposure to chromium caused decreases in gonad weight, GSI, and fecundity in Japanese medaka fish, *Oryzias latipes* [108]. After 10 days of exposure, Motta et al. [48] found that *Trematomus bernacchii* fecundity decreased due to cadmium accumulation. Environmental pollution altered the reproductive traits of wild female roaches (*Rutilus rutilus*) from the Seine River (France) [53]. They showed the inhibition of gonad maturation, reduced oocyte growth, and lower gonadal aromatase activity in the females of polluted waterbodies in the Seine River. Overall, these results highlight the presence of endocrine disruption in female roaches from the Seine River. Many studies have reported that heavy metals affect fish reproduction by lowering GSI [53], inhibiting hormone reproduction, reducing oocyte diameter [53,117], and changing reproductive behavior [118].

Our study detected bioaccumulation of Cd, Cr, Hg, and Pb in *C. punctata* of 5 freshwater habitats of Bangladesh, including the most studied Turag River and less studied Dharla and Atrai Rivers. The bioaccumulation level of these heavy metals, except mercury, was above the maximum acceptable concentration of FAO/WHO (2003). Our study estimated the length-weight relationship, condition factor, and reproductive biology of *C. punctata* collected from highly polluted freshwater habitats, viz., the Turag River, Karatoya River, and Ashura *Beel*, as well as collected samples from less polluted habitats, viz. the Atrai and Dharla Rivers of Bangladesh. The negative allometric growth pattern and poor wellness with *higher Cd, Cr, and Pb* bioaccumulation in *C. punctata of* Turag and Karatoya Rivers indicated an inverse relationship. In contrast, the lower level of bioaccumulation of these heavy metals in Atrai and Dharla Rivers *C. punctata* stocks showed higher growth and reproductive performances. We observed a similar inverse relationship between levels of heavy metal bioaccumulation and the reproductive performances of *C. punctata*. The higher cadmium and chromium concentrations are associated with a significant decrease in GSI, fecundity, and ova diameter in *C. punctata* of the Turag and Karatoya Rivers. The substantial biological effects of bioaccumulation of Cd, Cr, and Pb or a mixture of more contaminants may cause the lower growth and reproductive performance of studied *C. punctata* population. Our study also showed an increasing trend of mercury concentration in fish of freshwater habitats, especially in urban rivers, which may be due to the increasing number of battery-driven vehicles like autorickshaws in Bangladesh.

## 5 Conclusion

The study concluded that heavy metals contaminate the freshwater habitats of Bangladesh, which may cause higher levels of bioaccumulation in the aquatic organisms and impact their growth and reproduction. The study had limitations in sample collection and detection of heavy metal bioaccumulation from various organs, suggesting a need for further research to understand the effects of heavy metal bioaccumulation on fish health. Moreover, further studies, such as histological observation and biochemical and molecular investigation of the gonads and other organs, may be needed to provide evidence of heavy metal-induced growth and reproductive disturbances. The situation needs rapid recovery from the respective authority of the Bangladesh government to stop industrial, agricultural, and mining drainage disposing of the aquatic environment by establishing treatment units before direct drainage.

## CRediT authorship contribution statement

**Imran Parvez:** Writing – original draft, Methodology, Investigation, Funding acquisition, Conceptualization. **Sharmin Ahmed:** Methodology, Formal analysis. **Nazifa Tasnim:** Methodology, Formal analysis. **Rubaiya Pervin:** Visualization, Investigation. **Md Ashraful Alam:** Visualization, Investigation. **Md Nasir Khan:** Methodology, Formal analysis. **Yeasmin Ara:** Methodology, Formal analysis. **Harunur Rashid:** Writing – review & editing. **Siriporn Pradit:** Writing – review & editing, Supervision, Funding acquisition, Conceptualization.

## Funding

This work was supported by 10.13039/501100004508Prince of Songkla University and ministry of higher education, science, research and innovation under the Reinventing University Project (Grant Number REV65009) and also by the special allocation of 10.13039/501100001407Ministry of Science and Technology (MOST), government of Bangladesh (Grant number: BS 132, FY2021-2022).

## Declaration of competing interest

The authors declare that they have no known competing financial interests or personal relationships that could have appeared to influence the work reported in this paper.
